# Editorial for the Special Issue Entitled “Carbon-Based Quantum Dots”

**DOI:** 10.3390/nano13091465

**Published:** 2023-04-25

**Authors:** Su Chen

**Affiliations:** State Key Laboratory of Materials-Oriented Chemical Engineering, College of Chemical Engineering, Nanjing Tech University, 5 Xin Mofan Road, Nanjing 210009, China; chensu@njtech.edu.cn

Carbon-based quantum dots, also known as carbon dots (CDs), are a unique class of carbon-based nanoparticles with sizes below 10 nm. Such nanomaterials have garnered increasing attention owing to their superior properties, such as unique fluorescence and phosphorescence, low toxicity, excellent biocompatibility, ease of surface functionalization, and inexpensive production [1,2]. These desirable characteristics highlight the potential benefits of CDs in various applications, including optoelectronic devices, anti-counterfeiting, detection and sensors, photocatalysis, biomedicine applications, etc. [3,4]. Although research on CDs has made significant progress, there are still many challenges in developing effective synthesis and assembly strategies, preparing high-performance CDs and multifunctional nanocomposites with integrated characteristics, researching other properties besides optical properties, and expanding the application of CDs. These avenues of research inspired this Special Issue entitled “Carbon-Based Quantum Dots”. Several studies related to the synthesis and assembly, properties, and potential applications of CDs have been published in this collection, and they are briefly outlined below.

Recently, CDs with other properties beyond their optical properties have raised increasing interest among researchers. Various surface functional groups of CDs facilitate their surface functionalization and composition with inorganic, organic, polymeric, and biologically active substances. Benefiting from the unique reinforcement effects of CDs on the material matrix, CDs provide significant engineering application potential [5]. One of the contributions is focused on the investigation of CDs in terms of enhancing the mechanical properties and biocompatibility of composites [6]. Syntheses of forsythia-derived CDs (F-CDs) and their incorporation into nylon-11 nanofibers to improve mechanical properties and biocompatibility were reported. The prepared F-CDs with rich surface functional groups could be well embedded in nylon-11 nanofibers through electrostatic spinning, resulting in significantly enhanced mechanical properties of nylon-11/F-CD nanofiber mats. In addition, nylon-11/F-CD nanofiber mats exhibited excellent cellular compatibility with L929 fibroblasts, with a cell viability rate of 96%. These findings may serve as a reference for the development of various CD-embedded nanofiber mats with good mechanical properties and biocompatibility.

Biomass CDs have garnered great attention owing to their superior advantages such as green technology, low toxicity, cost efficiency, and simple preparation [7]. In one study, a natural plant polyphenol (tannic acid) was used as a carbon source to hydrothermally synthesize red, green, and blue tunable luminescent CDs by changing the pH value of the reaction system [8]. In another study, nitrogen-doped multicolor CDs, including green, yellow–green, and pink luminescent CDs, were successfully synthesized using kiwi fruit juice as a carbon source by adding different solvents such as ethanol, ethylenediamine, and acetone for ultrasonic treatment [9]. The potentials of these CDs for applications such as fluorescent inks, fluorescence sensors, and logic gate operations were also discussed. The synthesis of these biomass CDs provides more possibilities for the application of environmentally friendly CDs in fields such as environmental monitoring and biomedicine.

Besides the unique advantages of CDs, building multifunctional nanocomposites with integrated characteristics by combining CDs with other functional materials is a particularly interesting area of research [10]. In this regard, Stepanova et al. [11] prepared magneto-luminescent nanocomposites (MLNCs) via the microwave treatment of carbon dots and magnetic nanoparticle precursors. The prepared nanocomposites exhibited both magnetic and PL properties, excellent solubility in water, adjustable PL, high photostability, and good cytocompatibility. Owing to these merits, they are considered promising candidates for bioimaging and therapeutics. In another contribution, Shen et al. [12] constructed multifunctional nanocomposites by combining quantum dots or carbon quantum dots with smart gels using a simple and rapid frontal polymerization method. By using self-healing quantum-dot-loaded gels as building units, complex structures with different fluoresces were constructed. This strategy provides a new perspective on the construction of various fluorescent components through self-healing assembly and potentially promotes future applications of self-healing gels using this novel strategy.

This Special Issue summarizes the latest advances related to the synthesis and assembly, unique properties, and potential applications of CDs. Some of these methods have practical significance for the preparation of high-performance environmentally friendly CDs and multifunctional nanocomposites. The selected papers amassed in this collection cover different aspects of CDs, and these novel contributions highlight some points of reference and inspiration for researchers in this field, thus promoting the development and application of CDs.
